# Association between telomere length and atopic dermatitis among school‐age children

**DOI:** 10.1002/clt2.70066

**Published:** 2025-05-28

**Authors:** Hsin‐Yi Huang, Kun‐Hua Sheen, Chi‐Yen Hung, Ju Chang‐Chien, Shih‐Ling Wang, Chia‐Hua Ho, Hui‐Ju Tsai, Tsung‐Chieh Yao

**Affiliations:** ^1^ Division of Allergy, Asthma, and Rheumatology Department of Pediatrics Chang Gung Memorial Hospital Taoyuan Taiwan; ^2^ Institute of Environmental and Occupational Health Sciences National Yang Ming Chiao Tung University Taipei Taiwan; ^3^ Department of Medical Science National Tsing Hua University Hsinchu Taiwan; ^4^ Department of Traditional Chinese Medicine Chang Gung Memorial Hospital Taoyuan Taiwan; ^5^ Department of Medicine Chang Gung University College of Medicine Taoyuan Taiwan; ^6^ Department of Family Medicine Chang Gung Memorial Hospital Taoyuan Taiwan; ^7^ Institute of Population Health Sciences National Health Research Institutes Zhunan Taiwan

**Keywords:** atopic dermatitis, children, telomere length

## Abstract

**Background:**

Atopic dermatitis is a common chronic skin disease in children. Whether telomere length is associated with atopic dermatitis remains unclear. This population‐based case‐control study aimed to investigate the association between telomere length and atopic dermatitis in school‐age children.

**Methods:**

In this cross‐sectional analysis, we included 1084 singleton term‐born children (608 males; mean age 6.4 years) from the Longitudinal Investigation of Global Health in Taiwanese Schoolchildren cohort. Telomere length was measured using quantitative real‐time polymerase chain reaction, log‐transformed and was analyzed in quartiles. The main outcome was atopic dermatitis defined as having physician‐diagnosed atopic dermatitis and the presence of atopic dermatitis in the last 12 months. Regression analyses were used to assess the relationship between telomere length and atopic dermatitis.

**Results:**

Telomere length was significantly inversely associated with childhood atopic dermatitis after adjusting for child's age, sex, overweight or obesity, birth season, childhood allergic diseases, environmental tobacco smoke, parental history of allergic diseases, parental educational level, and breastfeeding status (*p*__trend_ = 0.01). Specifically, when telomere length was classified into quartiles, children in the shortest (fourth) telomere length quartile had a 1.88‐fold higher probability of atopic dermatitis compared to those in the longest (first) quartile (95% confidence interval: 1.13–3.14). Stratified analyses showed that the associations were stronger in males and non‐breastfed children, with no significant associations observed in females or breastfed children.

**Conclusion:**

This study provides new evidence suggesting an association between shorter telomere length and atopic dermatitis in school‐age children.

## 1 INTRODUCTION

Human telomeres are nucleoprotein complexes composed of tandemly repeated DNA sequences, which play a crucial role in protecting chromosomes and maintaining chromosomal stability.^1^ However, telomere length shortens with each round of cell division or as a result of DNA damage;^1^ thus, telomere length has been considered a biomarker associated with aging and chronic diseases.^2^ While the relationship between telomere length and various human diseases, such as type II diabetes, cardiovascular diseases, and cancer etc., has been documented,^3^, ^4^, ^5^, ^6^ limited studies have investigated the association between telomere length and atopic dermatitis, particularly in pediatric populations.

Atopic dermatitis is a common chronic and recurrent skin disease that often causes inflammation, irritation, and redness of the skin. The prevalence of atopic dermatitis has been rising worldwide, including in Taiwan,^7^, ^8^, ^9^, ^10^, ^11^ posing a substantial healthcare burden.^12^ Some risk factors, such as genetic factors, history of food allergy, and environmental factors, have been found to be associated with the development and/or severity of atopic dermatitis.^13^ Previous reports have documented that chronic inflammation is related to telomere length shortening.^14^ It is possible that changes in telomere length may impact the risk of atopic dermatitis. However, to date, whether telomere length is associated with childhood atopic dermatitis has barely been investigated.

Previous studies have examined the relationship between telomere length and atopic dermatitis,^15^, ^16^ but the results were inconsistent. For example, Wu et al. found that 32 adults with atopic dermatitis had shorter telomere lengths compared to those in the control group.^15^ However, Suh et al. reported no difference in telomere length between 68 infants with and without atopic dermatitis.^16^ It remains unknown regarding the relationship between telomere length and childhood atopic dermatitis in large cohort studies. In this study, we investigated the association between telomere length and atopic dermatitis in a cohort of Asian school‐age children.

## 2 METHODS

### 2.1 Study subjects

**FIGURE 1:**
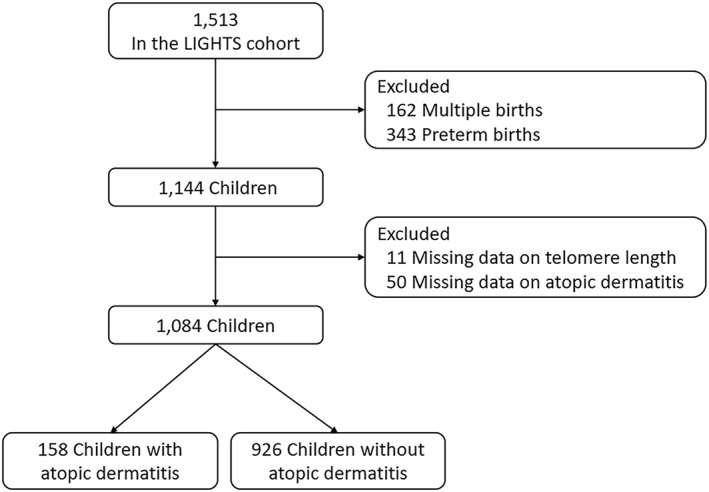
Flow chart for the enrollment process of study subjects.

This study included 1084 singleton term‐born children, a subset of 1513 children participating in the Longitudinal Investigation of Global Health in Taiwanese Schoolchildren (LIGHTS) study. The study children were born during 2010–2011 and attended a follow‐up visit in the Chang Gung Memorial Hospital during 2016–2018. Detailed information about data collection in the LIGHTS cohort was described elsewhere.^17^, ^18^, ^19^, ^20^, ^21^, ^22^, ^23^, ^24^, ^25^ Particularly, we excluded participants with multiple births (*n* = 162), preterm births (*n* = 343), missing data on telomere length (*n* = 11), and missing data on atopic dermatitis (*n* = 50), a total of 1084 study children were included in the subsequent analysis (Figure 1). Parents of the study children answered pre‐designed questionnaires. The collected data included participants' general health information, demographic and clinical data. The weight and height of each participant were measured by trained medical staff in the Chang Gung Memorial Hospital. Body mass index (BMI) was calculated based on weight (kg)/height squared (m^2^). Peripheral blood samples were collected for measuring telomere length. The study procedures were approved by the Institutional Review Board of Chang Gung Medical Foundation (No. 201600334A3). The parents of the study children provided written informed consent.

### 2.2 Measurement of telomere length

Genomic DNA was extracted from peripheral blood samples using a DNA extraction kit (RBC Bioscience). DNA samples with an A260/A280 ratio of 1.8–2.0 were used for further assays. The genomic DNA samples were diluted to the same concentration (20 ng) before telomere length measurement. Quantitative real‐time polymerase chain reaction (qPCR) method was used to measure telomere length as described previously.^25^, ^26^ Relative telomere length for each sample was calculated as the T/S ratio defined as the ratio of telomere repeat copy number to single‐copy reference gene (*36B4*) copy number. The qPCR reactions were performed using iQ™ SYBR Green Supermix on a CFX96 Real‐Time System (BioRad). The thermal cycles for both telomeres and single gene started at 95°C for 10 min, followed by 30 PCR cycles (95°C for 30 s and 58°C for 30 s). Cycle threshold (Ct) values were used to calculate the T/S ratio according to the 2^−ΔΔCt^ method.^27^, ^28^ The specific primers used for telomere length determination were listed in Table S1. To ensure reproducibility, each sample was analyzed in duplicate, with an intra‐assay coefficient of variation (CV) threshold set at <3%. Samples exceeding this threshold were reanalyzed. Inter‐assay variation was calculated using DNA of K562 cells as a calibrator in all qPCR plates, with the inter‐assay CV equal to 1.46% for telomere length and 0.98% for *36B4* standard. Batch‐specific efficiency adjustments were applied to minimize variability across different qPCR plates, with mean efficiencies of 90.58% for telomere length and 84.47% for *36B4* standard.^25^, ^26^ As an additional indicator of measurement reliability, we calculated the intraclass correlation coefficient (ICC) to estimate the proportion of the total variance attributable to differences between participants.^29^, ^30^ The ICC for telomere length measurement was 0.93, indicating excellent reproducibility.

### 2.3 Definitions of atopic dermatitis

Data on atopic dermatitis were collected using a modified International Study of Asthma and Allergies in Childhood questionnaire, which was completed by the parents of participating children (with a mean age of 6.4 years) at the follow‐up visit. Parents of participating children were interviewed by a pediatrician at the same visit, and answered the following atopic dermatitis related questions: “Has your child ever had atopic dermatitis diagnosed by a physician?”; and “Has your child had symptoms of atopic dermatitis in the last 12 months?”. Cases were defined as children who had physician‐diagnosed atopic dermatitis and reported symptoms of atopic dermatitis in the last 12 months.

### 2.4 Covariates

All covariates were collected at the 2016–2018 follow‐up visit. Information on covariates was obtained from parent‐reported questionnaires. In detail, the covariates, including age (years), sex (males/females), overweight or obesity (yes/no), birth season (spring, summer, autumn, or winter), childhood allergic diseases (yes/no), environmental tobacco smoke (ETS) (yes/no), parental allergic diseases (yes/no), parental educational level (university or above versus high school or below), and breastfeeding longer than 6 months (yes/no), were adjusted in analytical models.^17^, ^18^, ^22^, ^25^ Childhood allergic disease was defined as physician‐diagnosed asthma, allergic rhinitis, or food allergy. Parental allergic disease was defined as physician‐diagnosed asthma, allergic rhinitis, or atopic dermatitis in either the mother, father, or both. Overweight or obesity was determined using age‐ and sex‐specific BMI cut‐off values for children aged 2–18 years suggested by the International Obesity Task Force.^31^, ^32^, ^33^

### 2.5 Statistical analysis

All analyses were conducted using the Statistical Analysis System, version 9.4 for Windows (SAS Institute). Telomere length data were log‐transformed to obtain approximate normality. We used the chi‐squared test and Fisher's exact test, separately, for categorical variables; and Student's *t*‐test for continuous variables to compare the difference between children with and without atopic dermatitis. We applied multiple logistic regression models to test the association of telomere length treated as a log‐transformed variable with atopic dermatitis, and reported the corresponding adjusted odds ratios (AORs) and 95% confidence intervals (CIs). We next categorized telomere length in quartiles, with the longest telomere length as the first quartile and the shortest telomere length as the fourth quartile. We treated the first quartile as the reference group and performed multiple logistic regression models to estimate the association between telomere length (as a categorical variable) and atopic dermatitis. We controlled for the covariates listed above in the models. We examined the dose‐response relationship using the Cochran‐Armitage trend test. We also assessed potential effect modification of sex, breastfeeding status, and parental educational level by including the product terms of sex × telomere length, breastfeeding status × telomere length, and parental educational level × telomere length, separately, in the models. Subgroup analyses were conducted and stratified by sex, breastfeeding status, and parental educational level. *p*‐values <0.05 were considered statistically significant.

## 3 RESULTS

### 3.1 Baseline characteristics of the study children

Table 1 presents the characteristics of 1084 singleton term‐born children in the study (608 males; mean age: 6.4 ± 0.4 years). Among those, 158 (14.6%) children had atopic dermatitis, whereas 926 (85.4%) children did not have atopic dermatitis (Table 1). The median telomere length and interquartile range were 1.87 (1.56–2.31) for all children, 1.78 (1.48–2.05) for children with atopic dermatitis, and 1.90 (1.57–2.33) for those without atopic dermatitis. Children with atopic dermatitis tended to have a higher proportion of parental allergic diseases than those without atopic dermatitis. No significant differences were observed between the two groups regarding age, sex, overweight or obesity, breastfeeding status, parental educational level, ETS, and birth season.

**TABLE 1:** Summary characteristics of 1084 children in the study.

Variable	Total (*n* = 1084)	With atopic dermatitis (*n* = 158)	Without atopic dermatitis (*n* = 926)	*p* value^a^ ^,^ ^b^
Age, mean (SD), y	6.4 (0.4)	6.4 (0.4)	6.4 (0.4)	0.21
Sex, male, *n* (%)	608 (56.1)	90 (57.0)	518 (55.9)	0.81
Body mass index, mean (SD)	15.8 (2.4)	16.0 (2.6)	15.8 (2.3)	0.25
Breastfeeding ≥6 months, *n* (%)	720 (66.5)	81 (13.9)	501 (86.1)	0.50
Environmental tobacco smoke, *n* (%)	417 (38.5)	57 (36.1)	360 (38.9)	0.50
Parental allergic diseases, *n* (%)				
Asthma	116 (10.8)	23 (14.8)	93 (10.1)	0.08
Allergic rhinitis	685 (63.3)	118 (74.7)	567 (61.3)	**0.001**
Atopic dermatitis	290 (26.8)	69 (43.7)	221 (23.9)	**<0.001**
Parental educational level, university or above, *n* (%)	945 (87.2)	140 (88.6)	805 (86.9)	0.56
Birth season, *n* (%)				
Spring	253 (23.3)	43 (27.2)	210 (22.7)	0.57
Summer	268 (24.7)	40 (25.3)	228 (24.6)	
Autumn	307 (28.3)	42 (26.6)	265 (28.6)	
Winter	256 (23.6)	33 (20.9)	223 (24.1)	
Childhood allergic diseases, *n* (%)				
Asthma	255 (23.6)	53 (33.5)	202 (21.9)	**0.002**
Allergic rhinitis	637 (59.0)	109 (69.0)	528 (57.3)	**0.01**
Food allergy	89 (8.3)	35 (22.2)	54 (5.9)	**<0.001**
Telomere length (median/IQR)	1.9/0.8	1.8/0.6	1.9/0.8	**0.01**

Abbreviations: IQR, interquartile range; SD, standard deviation.

^a^ *p*‐value is obtained from Student's *t*‐test (continuous variables) or chi‐squared test (categorical variables).

^b^ Bold values denote statistical significance at *p*‐value <0.05.

### 3.2 Association of telomere length with atopic dermatitis

**FIGURE 2:**
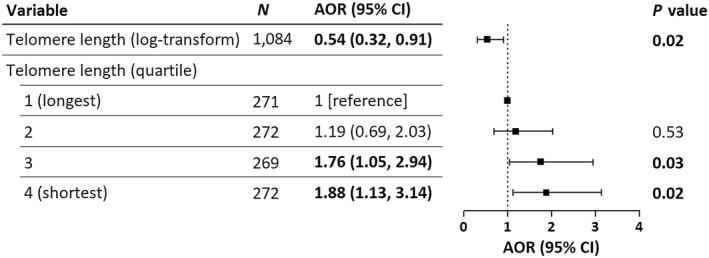
AORs and 95% CIs for associations between telomere length and atopic dermatitis in 1084 school‐age children. Adjusted for child's age, sex, overweight or obesity, birth season, childhood allergic diseases, environmental tobacco smoke, parental allergic diseases, parental educational level, and breastfeeding. Bold values denote statistical significance at *p*‐value <0.05. AORs, adjusted odds ratios; CIs, confidence intervals.

Figure 2 shows a significant association of telomere length with atopic dermatitis after adjusting for child's age, sex, overweight or obesity, birth season, childhood allergic diseases, ETS, parental allergic diseases, parental educational level, and breastfeeding status (*p*__trend_ = 0.01). Specifically, telomere length was inversely associated with atopic dermatitis, indicating that longer telomere length was associated with a decreased probability of atopic dermatitis (AOR: 0.54; 95% CI: 0.32–0.91). When categorized into quartiles, children in the fourth (the shortest) telomere length quartile had a 1.88‐fold higher probability of atopic dermatitis compared to those in the first (the longest) quartile (95% CI: 1.13–3.14).

### 3.3 Association of telomere length with atopic dermatitis stratified by child's sex, breastfeeding status and parental educational level

**FIGURE 3:**
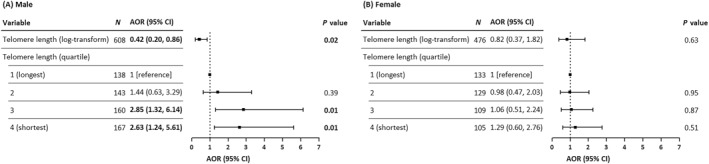
AORs and 95% CIs for associations between telomere length and atopic dermatitis stratified by sex. Adjusted for child's age, overweight or obesity, birth season, childhood allergic diseases, environmental tobacco smoke, parental allergic diseases, parental educational level, and breastfeeding. Bold values denote statistical significance at *p*‐value <0.05. AORs, adjusted odds ratios; CIs, confidence intervals.

We further conducted stratified analyses by child's sex, breastfeeding status, and parental educational level. Figure 3 shows a higher probability of atopic dermatitis among males with shorter telomere lengths. Specifically, males in the third and fourth quartiles of telomere length had an increased probability of atopic dermatitis compared to those in the first quartile (AOR: 2.85; 95% CI: 1.32–6.14 for the third quartile; and AOR: 2.63; 95% CI: 1.24–5.61 for the fourth quartile; Figure 3). No significant association was found between telomere length and atopic dermatitis among females.

**FIGURE 4:**
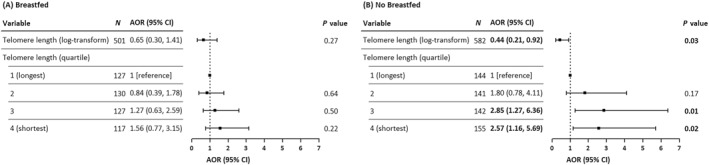
AORs and 95% CIs for associations between telomere length and atopic dermatitis stratified by breastfeeding. Adjusted for child's age, sex, overweight or obesity, birth season, childhood allergic diseases, environmental tobacco smoke, parental allergic diseases, and parental educational level. Bold values denote statistical significance at *p*‐value <0.05. AORs, adjusted odds ratios; CIs, confidence intervals.

When stratified by child's breastfeeding status, children who had not been breastfed and were in the third or fourth quartiles of telomere length had a higher probability of atopic dermatitis compared to those in the first quartile (AOR: 2.85; 95% CI: 1.27–6.36 for the third quartile; and AOR: 2.57; 95% CI: 1.16–5.69 for the fourth quartile; Figure 4). In contrast, no significant association between telomere length and atopic dermatitis was observed among children who had been breastfed. Table S2 shows that shorter telomere length was significantly associated with atopic dermatitis only among children in the group of parental educational level of high school or below (AOR: 17.66; 95% CI: 1.62–192.21) but not in the group of parental educational level of university or above.

However, no significant interactions were observed between sex and telomere length (*p* = 0.21), breastfeeding status and telomere length (*p* = 0.49), or parental educational level and telomere length (*p* = 0.77) in relation to atopic dermatitis. Therefore, the findings of stratified analyses should be interpreted with caution.

## 4 DISCUSSION

To our knowledge, this study is the first to examine the association between telomere length and atopic dermatitis in a population‐based cohort of school‐age children. Our results demonstrate an association between shorter telomere length and atopic dermatitis among 1084 Asian school‐age children. The association was more pronounced in males, children who were not breastfed during infancy, and children whose parental educational level was high school or below, whereas no such association was observed in females, breastfed children, or children whose parental educational level was university or above. These findings underscore the potential detrimental effect of shorter telomere length on the increasing probability of childhood atopic dermatitis.

In this study, we demonstrated an inverse association of telomere length with atopic dermatitis among school‐age children. A previous study in Denmark reported shorter telomere length in 32 adults with atopic dermatitis compared to 30 healthy controls.^15^ Our findings were in accordance with the Denmark study, and further indicated that the effect of telomere length on atopic dermatitis may emerge much earlier, manifesting as early as school age. However, research on the relationship between telomere length and childhood atopic dermatitis remains inconclusive. A study of 68 infants conducted by Suh et al. in Korea found no significant association between cord‐blood telomere length and atopic dermatitis in children at 1 year of age.^16^ Differences in age, timing of biospecimen collection, sample sizes, and study designs likely explain this discrepancy. Our findings suggest that telomere length may play a discernible role in the occurrence of atopic dermatitis later in childhood, warranting further research into how telomere dynamics contribute to disease risk over time.

Several factors may contribute to the observed association between shorter telomere length and atopic dermatitis. For example, Wu et al. reported increased telomerase activity in atopic dermatitis patients, suggesting that chronic inflammation stimulates immune cells led to extensive cell division and accelerated telomere attrition.^15^ Additionally, Omata et al. found elevated oxidative stress in children with atopic dermatitis,^34^ which may contribute to telomere length shortening. These biological mechanisms may partially explain the findings in the present study. Further research is needed to clarify the role of telomere length shortening in the pathogenesis of atopic dermatitis.

Stratified analyses revealed that shorter telomere length was associated with an increased probability of atopic dermatitis in males but not in females. Although the exact mechanisms underlying this sex‐specific effect remain unclear, biological differences between males and females may play a role in modulating the effect of telomere length shortening on atopic dermatitis in school‐age children. Therefore, further studies are needed to validate this observed sex difference and elucidate the underlying mechanisms. The stratified analyses also showed that non‐breastfed children with shorter telomere lengths had an increased probability of atopic dermatitis, whereas no association was found among breastfed children. Numerous studies have documented the anti‐inflammatory and immune‐protective effects of breastfeeding.^35^, ^36^ Exposure to oxidative stress and inflammation is linked to accelerated telomere shortening in adults.^37^ Previous studies also reported that exclusive breastfeeding at 4–6 weeks of age may influence telomere length in the preschool years, potentially due to its protective immunological effects.^38^ Further investigations are needed to explore how breastfeeding may mitigate telomere shortening and offer further insights into this relationship.

The strengths of this study included a large population‐based sample of 1084 school‐age children, objective measurements of telomere length, and comprehensive data on relevant covariates. However, some limitations should be considered. First, the data from parental‐report questionnaires were obtained retrospectively and might be subject to recall bias. Secondly, telomere length was measured cross‐sectionally, which limits our ability to observe telomere changes over time. Longitudinal studies are needed to confirm and extend the findings from this study. Third, this is a cross‐sectional analysis, which limits the ability to establish causal or temporal relationships and address the issue of reverse causality. Fourth, the study participants were limited to an Asian population. Additional research is needed to determine the generalizability of these observed results to other populations.

In conclusion, this study provides new evidence suggesting an association between shorter telomere length and atopic dermatitis in Asian school‐age children. These findings highlight the potential adverse effect of telomere shortening on atopic dermatitis. Further studies would be valuable in exploring whether telomere length could serve as a potential biomarker for the development, severity, and progression of atopic dermatitis and related atopic diseases.

## AUTHOR CONTRIBUTIONS

**Hsin‐Yi Huang**: Writing—original draft; methodology; data curation; formal analysis; investigation; visualization; project administration. **Kun‐Hua Sheen**: Writing—original draft; methodology; data curation; formal analysis; investigation; visualization. **Chi‐Yen Hung**: Writing—review and editing. **Ju Chang‐Chien**: Writing—review and editing; methodology; project administration. **Shih‐Ling Wang**: Methodology; project administration. **Chia‐Hua Ho**: Writing—review and editing. **Hui‐Ju Tsai**: Conceptualization; funding acquisition; supervision; writing—review and editing; validation; investigation; software. **Tsung‐Chieh Yao**: Conceptualization; funding acquisition; supervision; writing—review and editing; resources; validation; investigation; software.

## CONFLICT OF INTEREST STATEMENT

The authors declare no conflicts of interest.

## Supporting information

Supporting Information S1

## Data Availability

The data that support the findings of this study are available on request from the corresponding author. The data are not publicly available due to privacy or ethical restrictions.
