# Supplementary material for: Association between telomere length and atopic dermatitis among school‐age children
Source: Clin Transl Allergy. 2025 May 28;15(6):e70066. doi: 10.1002/clt2.70066 (PMC12119238; doi:10.1002/clt2.70066)
Supplement: Supplementary file 1 — Supporting Information S1 [file CLT2-15-e70066-s001.docx]

**Supporting Information**

**Association between telomere length and atopic dermatitis among school-age children**

Hsin-Yi Huang, MS^1,2,†^, Kun-Hua Sheen, BS^3,†^, Chi-Yen Hung, MD^4^, Ju Chang-Chien, PhD^1,5^, Shih-Ling Wang, MS^1,5^, Chia-Hua Ho, MD^6^, Hui-Ju Tsai, MPH, PhD ^3,7,*^, Tsung-Chieh Yao, MD, PhD ^1,5,*^

^1^Division of Allergy, Asthma, and Rheumatology, Department of Pediatrics, Chang Gung Memorial Hospital, Taoyuan, Taiwan

^2^Institute of Environmental and Occupational Health Sciences, National Yang Ming Chiao Tung University, Taipei, Taiwan

^3^Department of Medical Science, National Tsing Hua University, Hsinchu, Taiwan

^4^Department of Traditional Chinese Medicine, Chang Gung Memorial Hospital, Taoyuan, Taiwan

^5^Department of Medicine, Chang Gung University College of Medicine, Taoyuan, Taiwan

^6^Department of Family Medicine, Chang Gung Memorial Hospital, Taoyuan, Taiwan

^7^Institute of Population Health Sciences, National Health Research Institutes, Zhunan, Taiwan

^†^These authors contributed equally.

^*^Corresponding authors

**Table S1.** Primers used for determining human telomere length.

| **Gene name** | **Forward Primer Sequence (5’-3’)** | **Reverse Primer Sequence (5’-3’)** |
| --- | --- | --- |
| **For measurement of telomere length** | | |
| *Telomere* | ACACTAAggTTTgggTTTgggTTTgggTTTgggTTAgTgT | TgTTAggTATCCCTATCCCTATCCCTATCCCTATCCCTAACA |
| *36B4* | CAgCAAgTgggAAggTgTAATCC | CCCATTCTATCATCAACgggTACAA |

**Table S2.** AORs and 95% CIs for associations between telomere length and atopic dermatitis stratified by parental educational level.

|  | **Parental educational level** | | | | | |
| --- | --- | --- | --- | --- | --- | --- |
|  | **University or above** | | | **High school or below** | | |
|  | ***N*** | **AOR (95% CI)** | ***p*-value** | ***N*** | **AOR (95% CI)** | ***p*-value** |
| Telomere length (log-transform) | 945 | 0.64 (0.37, 1.11) | 0.12 | 139 | **0.10 (0.02, 0.72)** | **0.02** |
| Telomere length (quartile) |  |  |  |  |  |  |
| 1 (longest) | 241 | 1 [reference] | - | 30 | 1 [reference] | - |
| 2 | 229 | 1.16 (0.66, 2.03) | 0.61 | 43 | 4.10 (0.39, 42.75) | 0.24 |
| 3 | 230 | 1.59 (0.93, 2.71) | 0.09 | 39 | 9.64 (0.90, 102.82) | 0.06 |
| 4 (shortest) | 245 | 1.58 (0.93, 2.69) | 0.09 | 27 | **17.66 (1.62, 192.21)** | **0.02** |

Adjusted for child’s age, sex, overweight or obesity, birth season, childhood allergic diseases, environmental tobacco smoke, parental allergic diseases, and breastfeeding.

Bold values denote statistical significance at *p*-value <0.05.

AORs, adjusted odds ratios; CIs, confidence intervals.
